# Prevalence of chronic kidney disease in the Lazio region, Italy: a classification algorithm based on health information systems

**DOI:** 10.1186/s12882-020-1689-z

**Published:** 2020-01-28

**Authors:** Claudia Marino, Pietro Manuel Ferraro, Matteo Bargagli, Silvia Cascini, Nera Agabiti, Giovanni Gambaro, Marina Davoli

**Affiliations:** 1 Department of Epidemiology Lazio Regional Health Service, Via Cristoforo Colombo, 112, 00147 Roma, Italy; 2 U.O.C. Nefrologia, Fondazione Policlinico Universitario A. Gemelli IRCCS, Largo Agostino Gemelli, 8, 00168 Roma, Italy; 3 Università Cattolica del Sacro Cuore, Roma, Largo Francesco Vito, 1, 00168 Roma, Italy; 4 Department of Medicine, Renal Unit, Division of Nephrology and Dialysis, University of Verona, Piazzale Ludovico Antonio Scuro 10, 37134 Verona, Italy

**Keywords:** Administrative data, Chronic kidney disease, Prevalence, Diagnostic algorithm, Health administrative datasets

## Abstract

**Background:**

Estimating CKD prevalence is difficult. Information on CKD prevalence is rather scanty in Italy and available figures come from surveys in selected geographical areas. Administrative data have been already demonstrated to be an effective tool in estimating the epidemiological burden of diseases, however there is limited experience in literature as far as CKD is concerned.

**Methods:**

The aim of this study is to develop an algorithm based on regional Health Administrative Databases to identify individuals with CKD and provide estimates of disease prevalence in Lazio Region (Italy); about 5.500.000 inhabitants in 2017. A population-level analysis based on a record-linkage strategy using data from Health Administrative Databases has been applied in Lazio Region. CKD cases were identified between January 1, 2012 and December 31, 2017 using Outpatient Specialist Service Information System, Hospital Discharge Registry, Ticket Exemption Registry and Drug Dispensing Registry. Age-specific and standardized prevalence rates were calculated by gender. CKD cases were classified as higher and lower severity.

**Results:**

The algorithm identified 99,457 individuals with CKD (mean age 71 years, 55.8% males). The exclusive contributions of each regional source used were: 35,047 (35.2%) from Outpatient Specialist Service Information System, 27,778 (27.9%) from Hospital Discharge Registry, 4143 (4.2%) from Ticket Exemption Registry and 463 (0.5%) from Drug Dispensing Registry; 5.1% of cases were found in all databases. The standardized prevalence rate at December 31, 2017 was 1.76, 2.06% for males and 1.50% for females. The prevalence increased with age, rising from 0.33% (age 0–18) up to 14.18% (age 85+) among males and from 0.25% up to 8.18% among females. The proportion of CKD individuals with lower severity disease was 78.7% in both genders.

**Conclusions:**

The proposed algorithm represents a novel tool to monitor the burden of CKD disease, that can be used by the regional government to guide the development and implementation of evidence-based pathways of care for CKD patients. The high prevalence of people with CKD of lower severity should be carefully considered in order to promote diagnosis and optimal management at early stages.

## Background

Chronic kidney disease (CKD) is a common chronic condition, which may often lead to renal failure. It increases the risk of cardiovascular complications and is linked to a variety of signs and symptoms, evidence of multi-organ dysfunction such as chronic anemia, inflammation, mineral bone disease and sarcopenia [1]. A recent meta-analysis showed that CKD prevalence is probably underestimated and higher than that of diabetes; in fact, worldwide CKD prevalence was 13.4% for stages 1–5 and 10.6% for stages 3–5 [2, 3]. An Italian epidemiological study with data at national level reported prevalence rates of 7.5% among males and 6.5% among females for the age category 35–79 years [4]. Therefore, CKD encompasses a variety of disorders and represents a true major public health burden [3]. The economic burden of CKD is substantial. Disease stage is predictive of higher costs, with particularly burdensome expenditures for end-stage renal disease (ESRD) due to renal replacement therapy (dialysis or transplantation) [5]. Although most of the costs per patient in the CKD population is related to ESRD, earlier stages also generate costs, mainly by inducing cardiovascular events [6]. Actually, costs in the earlier stages of CKD before dialysis are less investigated [7].

In Italy, the estimated annual cost of pre-dialysis patients is € 7422 for CKD stage IV and € 8971 for stage V. The costs are significantly higher for ESRD patients: the actual cost per year for dialysis patients is € 29,800 for those on peritoneal dialysis and € 43,800 for those on hemodialysis [8, 9]. Indirect costs should be added. Based on these data, it has been supposed that delaying the progression from CKD stage III to IV for 10% of affected patients for a 5-year period will save up to € 2.5 billion [10]. Hence, prevention, early diagnosis, management and care for people with CKD have high impact on health care programs in terms of direct and indirect costs. A working national or regional recognition program for CKD could allow to establish an early diagnosis, reducing health care costs and improving quality of life for the patients. Currently, a system with such characteristics is missing in Italy and, to the best of our knowledge, in Europe. Since it is difficult to assess CKD in large-scale models based on laboratory parameters only, there is actually increasing interest in the use of administrative data for epidemiological purposes.

Administrative data have been already demonstrated to effectively estimate prevalence of cardiovascular disease and diabetes with high sensitivity and specificity [11, 12]. In order to achieve the same results, a systematic review analyzed 13 administrative database-coding algorithms for kidney disease, but it found low sensitivity and variable positive predictive value regarding CKD [13].

In Italy, a recent systematic review highlighted the paucity of works conducted to identify people with CKD based on routinely collected data [14]. CKD has multiple causes and clinical pictures, with increasing levels of severity. While more advanced disease can be captured in the administrative databases by intercepting the prescription of specific drugs and procedures, milder stages of disease need to be assessed through more complex, integrated multi-sources algorithms. Unfortunately, to the best of our knowledge no such approach has ever been applied.

There is a need to measure the complete burden of CKD and develop procedures based on administrative data, in order to monitor temporal and geographic variation in occurrence, to evaluate quality of care for these patients, and to support the implementation of new organizational integrated care model [15]. In Italy, regional registries on dialysis exist, and there is a coordination effort at national level to describe and monitor this stage of the disease (https://ridt.sinitaly.org/). Moreover, the Italian Ministry of Health in 2017 promoted the institution of the national CKD registry – including all stages - to describe the epidemiology, to monitor the quality of care and health outcomes, and to prevent the incidence of the most severe – and costly – stages of this disease [10].

## Methods

The aim of this study is to develop an algorithm based on health information systems, to identify people with CKD and to provide prevalence estimates in Lazio, a highly-populated region in central Italy. Such approach could potentially be extended in other Italian regions.

### Data sources

#### Health administrative data

Different health information systems were used to identify the population with CKD in the Lazio region (over 5,700,000 residents). The Hospital Discharge Registry (HDR) routinely collects data from all regional hospitals, including information on patients’ demographic characteristics, up to six discharge diagnoses and up to six hospital procedures codes according to the International Classification of Disease-Ninth Revision-Clinical Modification (ICD-9-CM). HDR also collects data on inter-regional mobility, all hospitalizations of Lazio residents that occur outside the region. The Ticket Exemption Registry (TER) includes data on all residents who are entitled to co-pay fee exemption for some particular conditions, e.g. disability, chronic diseases, low income or old age. The Outpatient Specialist Service Information System (OSSIS) collects data from outpatient clinics (e.g. whether the participant underwent visits, specialized diagnostic-instrumental services and laboratory analyses). The Drug Dispensing Registry (PHARM) collects individual records for each drug prescription that is dispensed from public and private pharmacies and by hospital at discharge. Drugs are identified by the national drug register code, which refers to the International Anatomical Therapeutic Chemical Classification System (ATC). Individual patient data and date of dispensing are reported for each prescription. The Regional Register of Causes of Death lists the causes of death, coded according to ICD-9 revision, for all deaths of citizens residing in the region. Finally, the Regional Health Assistance File contains the history of cancellation and registrations of the residence for each citizen ensured by the Regional Health Service. The Lazio Dialysis Registry (LDR) is a population-based registry that started in 1994. It collects detailed information on all patients undergoing chronic dialysis (e.g., those undergoing either hemodialysis or peritoneal dialysis for a period of at least 90 days). It supports the planning, management, control and evaluation of health care, as well as the study and scientific research activities in the medical, biomedical and epidemiological fields. All dialysis units of the Lazio region are requested by law to register the information on their patients and to update it every 6 months.

All residents of the Lazio region served by the public health service have a personal identification number recorded in all the regional healthcare databases. This individual identifier provides the key to link all regional databases and allows to identify individuals uniquely within the regional health system.

### Study population

#### Case definition criteria

To identify cases with CKD at December 312,017 we used HDR, TER, OSSIS and (PHARM) during the time from January 012012 to December 312,017.

From HDR, we selected all subjects with at least one hospital discharge (primary or secondary diagnosis) or procedures referred to codes reported in Table 1. To avoid selecting acute renal failure, records of hospitalization selected only with procedures 39.95 (hemodialysis) or 38.95 (venous catheterization for renal dialysis) and in which one of the diagnosis codes was 584.XX (acute renal failure) were eliminated. From TER, we selected all subjects registered at the date of prevalence with CKD (code: 025.585) or kidney transplantation (code: 025.V42.0). From OSSIS, we selected all subjects who had at least one record with codes listed in Table 1 and we considered only subjects who had undergone at least two nephrological visits or one nephrological visit plus at least one measurement of urine albumin or at least one prescription of the drugs listed in Table 1 (data source PHARM).

**Table 1:** Diagnosis (code = ICD-9-CM), procedures (code = ICD-9-CM), outpatient services (code = Regional codification) and drugs (code = ATC), name and code

Name	Code (diagnostic branch)
**The Hospital Discharge Registry (HDR)**	
Diagnosis	
Diabetes with renal manifestations	250.4X
Hypertensive chronic kidney disease	403.XX
Hypertensive heart and chronic kidney disease	404.XX
Chronic glomerulonephritis	582.XX
Nephritis and nephropathy not specified as acute or chronic	583.XX
Chronic kidney disease (ckd)	585.XX
Renal failure, unspecified	586.XX
Renal sclerosis, unspecified	587.XX
Disorders resulting from impaired renal function	588.XX
Cystic kidney disease	753.1X
Chronic pyelonephritis	590.0X
Encounter for dialysis and dialysis catheter care	V56.X
Kidney replaced by transplant	V42.0
Procedures	
Hemodialysis	39.95
Peritoneal dialysis	54.98
Transplant of kidney	55.6X
Arteriovenostomy for renal dialysis	39.27
Creation of cutaneoperitoneal fistula	54.93
Revision of arteriovenous shunt for renal dialysis	39.42
Removal of arteriovenous shunt for renal dialysis	39.43
Venous catheterization for renal dialysis	38.95
Closed [percutaneous] [needle] biopsy of kidney	55.23
Complex outpatient services for	
Assess diagnosis of nephropathies	P583
Assess chronic kidney disease	P585A
Assess kidney transplant	P585B
Follow-up of kidney transplant patient	PV420
**Outpatient Specialist Service Information System (OSSIS)**	
Services	
First ambulatory specialist visit (nephrology)	89.7 (29)
Ambulatory specialist visit (nephrology)	89.01 (29)
Measurement of urine albumin	90.33.4
Definition of the haemodialysis or peritoneal dialysis scheme (nephrology)	89.03 (29)
Hemodialysis or hemodiafiltration	39.95.X
Peritoneal dialysis	54.98.X
Venous catheterization for renal dialysis	38.95
Creation of cutaneoperitoneal fistula (peritoneal catheter)	54.93
Debriding of peritoneal catheter	39.99.1
Removal of peritoneal catheter	97.82
Revision of peritoneal catheter	97.29.1
**Drug Dispensing Registry (PHARM)**	**ATC**
Erythropoietin	B03XA01
Darbepoetin alfa	B03XA02
Methoxy polyethylene glycol-epoetin beta	B03XA03
Polystyrene sulfonate	V03AE01
Sevelamer	V03AE02
Lanthanum carbonate	V03AE03
Sucroferric oxyhydroxide	V03AE05

All patients who satisfied at least one of the above conditions were selected. We excluded patients who died during the period or patients who did not reside in the Lazio region at December 312,017.

#### Identification of CKD cases with different levels of severity.

CKD cases were classified into two levels of severity. Higher severity cases were subjects who during the selection period had undergone at least one dialysis or kidney transplant or one hospitalization with diagnosis code of CKD stage IV or greater (ICD-9-CM diagnosis code: 585.4X, 585.5X,585.6X) or who had been prescribed at least one of the drugs listed in Table 1. Lower severity cases were considered all the remaining individuals in the prevalent population.

### Statistical analysis

#### Prevalence rates

The prevalence estimates were estimated at December 312,017, named prevalence date. Age- and gender-specific prevalence rates (× 100) were calculated by dividing the number of patients with CKD alive and resident at the prevalence date by the number of residents in the Lazio region. The age classes considered were 0–18, 19–44, 45–64, 65–74, 75–84, 85+. Additionally, we calculated standardized prevalence rates % (direct method) separately by gender and severity of CKD, using the Italian ISTAT population at December 312,017 as reference population. For all measures reported, 95% Confidence Intervals (95%CI) were estimated.

*Validity of the dialysis case ascertainment algorithm.*

The availability of the Lazio Dialysis Registry data gave us the opportunity to evaluate the validity of our algorithm in the identification of a sub-group out of the whole CKD population, namely those patients who received dialysis during the selection period. Patients undergoing chronic dialysis between January 012012 and December 312,017 alive and resident at the prevalence date were selected from the Lazio Dialysis Registry and were linked with the prevalent cases who had received dialysis treatments according to the algorithm. Considering Lazio Dialysis Registry cases as the gold standard for dialysis treatments, we calculated sensitivity, specificity, positive and negative predictive values of the algorithm [15, 16].

All analyses were performed using SAS Version 9.4. This study was carried out in full compliance with the current privacy laws. It was based on anonymous computer records from health information systems and did not require ethical approval.

## Results

**Fig. 1:**
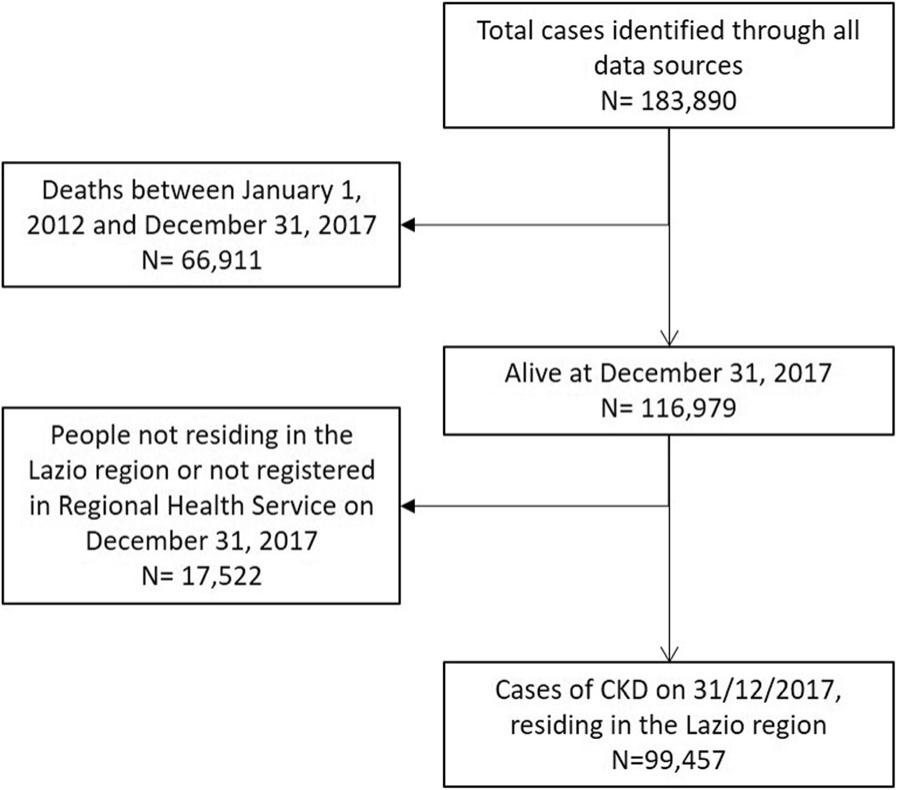
Flow-chart selection of Chronic Kidney Disease (CKD) prevalence cases at December 312,017, resident in the Lazio region

The algorithm identified a population with CKD of 99,457 individuals at December 312,017 (Fig. 1).

**Fig. 2:**
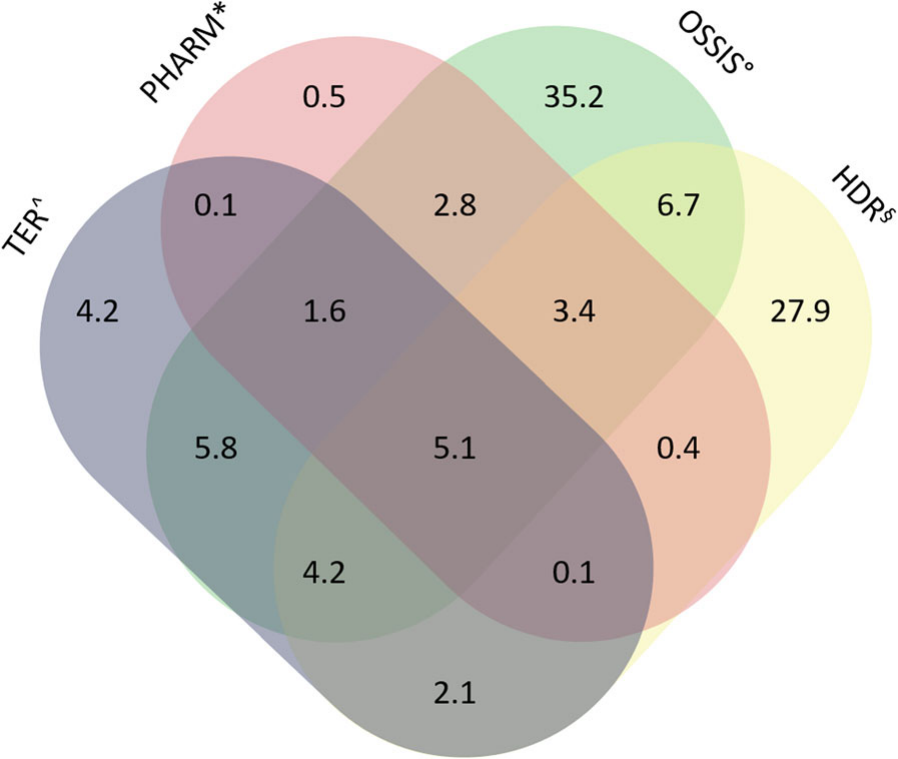
Percentage contribution of regional health information systems to chronic kidney disease cases identification. ^TER: Ticket Exemption Registry; * PHARM: Drug Dispensing Registry; °OSSIS: Outpatient Specialist Service Information System; ^§^HDR: The Hospital Discharge Registry

Males were 55.8% of the total, mean age was 70 years (standard deviation (SD) 17.7) for males and 72 years (SD 18.0) for females (*p*-value < 0.0001 for the difference between males and females). The exclusive contributions from each source to the identification of CKD cases were: 35,047 (35.2%) from OSSIS, 27,778 (27.9%) from HDR, 4143 (4.2%) from TER and 463 (0.5%) from PHARM; 5.1% of cases were found in all databases (Fig. 2).

The crude prevalence rate of CKD in the Lazio region was 1.76% (95%CI 1.75, 1.78), 2.06% for males and 1.50% for females. The prevalence increased by age group, from 0.33% (age 0–18) up to 14.18% (age 85+) in males and from 0.25% up to 8.18% in females (Table 2). The lowest male to female ratio was in age class 19–44 and the highest in age class 65–74 (1:14 and 1:83, respectively). The standardized prevalence was 2.35% (95% CI 2.33, 2.37) for males and 1.39% (95% CI 1.38, 1.40) for females, with a male to female ratio of 1:69 (Table 2).

**Table 2:** Age- and gender-specific prevalence per 100 population on December 312,017, Lazio region, Italy

Age class	Males					Females					Total					Rate M/F
	Cases	Pop^a^	Prev %	95% CI		Cases	Pop^a^	Prev %	95% CI		Cases	Pop^a^	Prev %	95% CI		
				Lower	Upper				Lower	Upper				Lower	Upper	
0–18	1604	490,339	0.33	0.31	0.34	1163	461,531	0.25	0.24	0.27	2767	951,870	0.29	0.28	1.30	1.30
19–44	2791	851,677	0.33	0.32	0.34	2487	866,373	0.29	0.28	0.30	5278	1,718,050	0.31	0.30	1.14	1.14
45–64	10,511	826,673	1.27	1.25	1.30	7346	900,610	0.82	0.80	0.83	17,857	1,727,283	1.03	1.02	1.56	1.56
65–74	13,302	281,274	4.73	4.65	4.81	8474	327,045	2.59	2.54	2.65	21,776	608,319	3.58	3.53	1.83	1.83
75–84	18,746	189,776	9.88	9.74	10.01	14,381	257,535	5.58	5.50	5.67	33,127	447,311	7.41	7.33	1.77	1.77
85+	8578	60,502	14.18	13.90	14.46	10,074	123,175	8.18	8.03	8.33	18,652	183,677	10.15	10.02	1.73	1.73
Total	55,532	2,700,241	2.06	2.04	2.07	43,925	2,936,269	1.50	1.48	1.51	99,457	5,636,510	1.76	1.75	1.37	1.37
STD Rate^b^			2.35	2.33	2.37			1.39	1.38	1.40			1.80	1.78	1.81	1.69

^a^Population of residents in the Lazio region and alive on the prevalence date (December 312,017)

^**b**^Prevalence*100 standardized to the Italian ISTAT population (December 312,017)

**Fig. 3:**
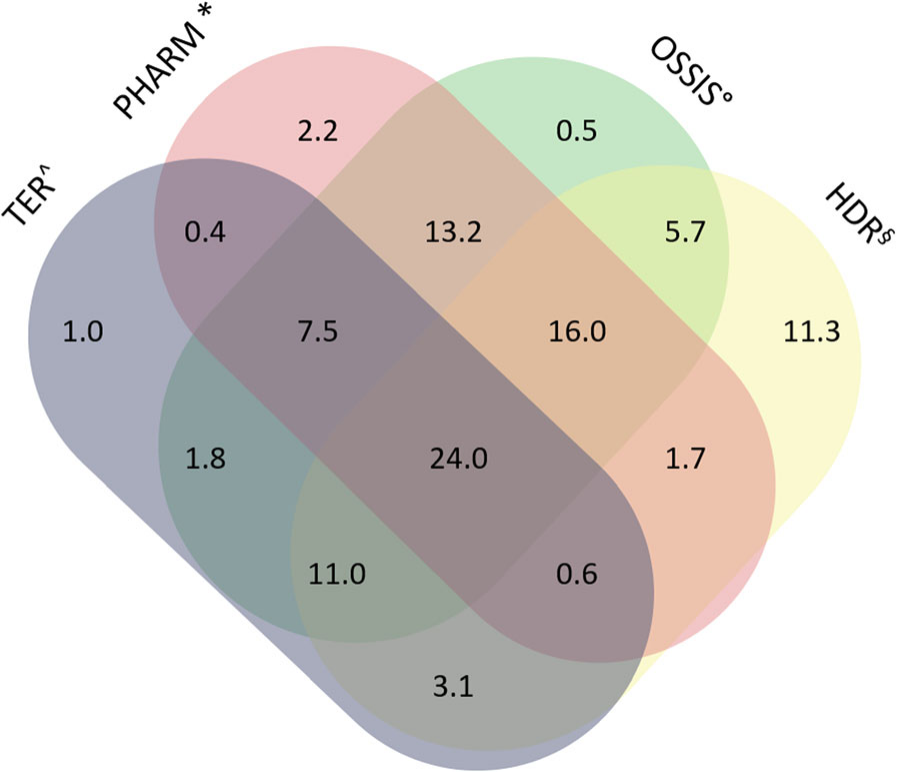
Percentage contribution of regional health information systems to higher severity chronic kidney disease cases identification. ^TER: Ticket Exemption Registry; * PHARM: Drug Dispensing Registry; °OSSIS: Outpatient Specialist Service Information System; ^§^HDR: The Hospital Discharge Registry

The algorithm identified 21,159 higher severity cases (21.3% of all CKD cases). Males were 55.8% of the total, and mean age for males and females was 71 (SD 15.3) and 74 (SD 15.8) years, respectively. The exclusive contributions from each source to the identification of higher severity CKD cases were: 116 (0.5%) from OSSIS, 2400 (11.3%) from HDR, 219 (1.0%) from TER and 463 (2.2%) from PHARM; 24.0% of cases were found in all databases (Fig. 3).

**Fig. 4:**
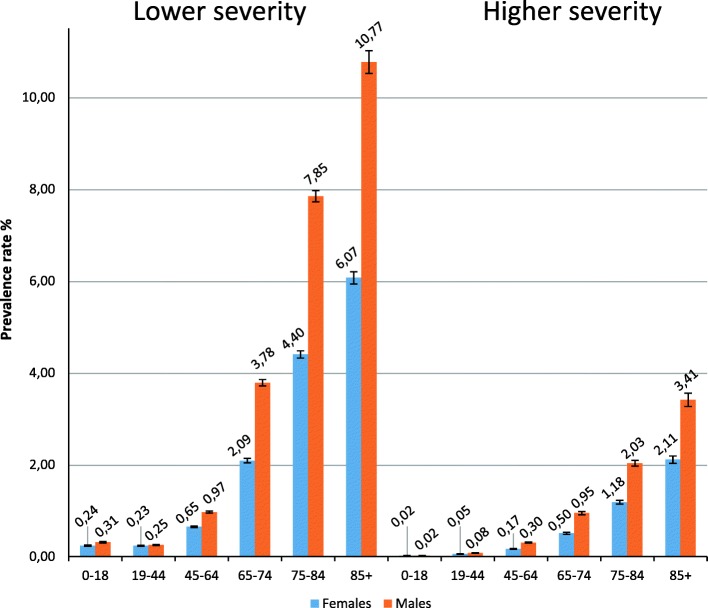
Chronic kidney disease prevalence rates per 100 population by age, gender and severity, Lazio region, Italy

For males, the standardized prevalence rate was 0.50% (95% CI 0.50, 0.51) for higher severity and 1.85% (95% CI 1.83, 1.87) for lower severity; the corresponding values for females were 0.29% (95% CI 0.29, 0.30) and 1.10% (95% CI 1.09, 1.11). Looking at age-specific prevalence, no difference was observed in the age class 0–18 in higher severity by gender. The prevalence of higher severity cases increased across age classes from 0.02% up to 3.14% (0–18, 85+ respectively) for males and from 0.02% up to 2.11% for females (Fig. 4).

The validity analysis on the ability of the algorithm to identify chronic dialysis patients showed sensitivity of 99.8%, specificity 99.9%, positive predictive value of 81.8% and negative predictive value 100.0%.

## Discussion

Our study represents the first experience in evaluating CKD in a large Italian region using a complex algorithm based on administrative data. It was based on multiple data collection from several databases and identified 99,457 individuals with CKD. The crude prevalence rate of CKD in the Lazio region was 1.76% (2.06% for males and 1.50% for females), with an increasing trend for higher age groups (up to 10.15% for patients with more than 85 years old). Higher severity cases were 21.3% of the entire CKD population.

Estimating CKD prevalence is rather complicated. In Italy, some studies managed to predict the exact prevalence of this condition only in certain geographical areas of the country. For instance, the INCIPE study showed a prevalence of 13.2% in the Italian northeastern population older than 40 years, but its methodology is difficult to apply for the entire country and requires dedicated funding and resources [17]. Newer research models tried to improve CKD detection by including general data. In fact, using information from the 2009 and 2010 Health Survey for England, a recent model was created to estimate and predict CKD prevalence among general population, through the collection of data regarding serum creatinine (response dichotomous variable: estimated glomerular filtration rate (eGFR) > or < 60 mL/min per 1.73m^2^), demographic variables (age, sex, ethnicity), area level variables (tenure, vehicle ownership, general health) and limiting long term illness. Therefore, serum creatinine was also needed in order to apply this multilevel small area synthetic estimation (ML-SASE) methodology [18].

To overcome this limitation, administrative data was considered for CKD prevalence analysis. This method is actually used by the US Renal Data System for CKD surveillance. This system defines patients with CKD through the presence of at least 1 ICD-9-CM diagnosis code from inpatient claims or at least 2 from outpatient claims or physician and supplier service claims for kidney disease [19].

As already mentioned, the successful use of administrative data for epidemiological purposes was fully achieved for diabetes, cardiovascular disease, and other diseases with relevant social and public heath impact [20–22] but it is more complex for CKD. In fact, a systematic review, including 25 observational studies from 13 administrative databases, used validated renal diagnostic codes and procedures for AKI and CKD to evaluate kidney disease against the reference laboratory standard [13]. Authors found low sensitivity and variable positive predictive value regarding CKD alone (41 and 78% respectively), albeit a better result was shown for dialysis only. To confirm these relatively poor results, Ronksley et al. examined the efficacy of an algorithm derived from administrative data to establish the epidemiology of CKD in Alberta, compared to eGFR. They used data from physician billing and hospital discharge abstracts, resulting in low sensitivity and positive predictive value when compared to clinical data (sensitivity of 19.4%, specificity of 97.2%, Positive Predictive Value of 60.1% and Negative Predictive Value of 84.8%) [23].

Health administrative databases have been widely used in Italy, as in many other countries, in the last decades for epidemiological purposes. The large amount of clinical information included in these datasets allow identification of acute and chronic health conditions. Disease-specific case-identification algorithms that combine information coded in multiple databases at a population level have been developed for many clinical conditions in the last years [24]. The systematic approach, the coverage at population level, the standardized methodology of data collection, and the large numbers are the main advantages of health information systems in order to describe the epidemiological burden of diseases and monitor the quality of a care. However, the use of these datasets have some limitations. Completeness and accurateness of data collected may be not adequate leading to potential misclassification of the case and its severity. Moreover, the heterogeneity in the quality of coding across different institutions, for example between private and public hospitals, may lead to difficulty in interpreting the variability in the occurrence of the disease. In our study, to identify CKD cases we used hospital discharge diagnoses, prescriptions of medications, disease-specific ticket exemption and drug prescriptions that all have a good level of good quality and completeness [20–22].

To compare our main results with the existing Italian literature about CKD epidemiology, we analyzed data from three major studies where CKD was defined, according to KDIGO definition, by evaluating eGFR and albumin excretion rate. The INCIPE study evidenced an age- and gender-adjusted CKD prevalence among the general population older than 40 years of northeastern Italy of 13.2% [17]. The GUBBIO study (central Italy) was conducted on 4574 subjects in the age range 18–95 years and highlighted a prevalence of CKD stage 3–5 of 5.7% for men and 6.2% for women [25]. These studies included only regional population of Italy and since CKD is a multifactorial heterogeneous condition, it is not possible to match their results with other regional datasets. On the contrary, the CARHES study analyzed a better geographically distributed Italian population composed of 7552 individuals aged between 35 and 79 years, resulting in a prevalence of 7.05% (7.54% for men and 6.54% for women). More specifically, the CKD prevalence of central Italy region was lower (5.60%) [4].

In our case, using administrative data, we estimated in Lazio, a central region of Italy, a CKD prevalence for age 45–84 of 2.62%. Although CKD in central regions of Italy seems to be less common, these numbers are still lower than expected based on the available epidemiology data [4]. On the other hand, in ≥85 years old people, CKD was much more common (prevalence of 10.15%), consistent with published data [2]. A possible explanation for these results is related to the data used by the algorithm, since they were available only for patients who resorted to at least one of the regional health systems in a 5 years period, reflecting more severe stages of CKD that actual led to access to care. In fact, it is less probable for patients with CKD stages 1–2 to attend medical services due to their CKD condition compared with CKD stages 3–5 subjects. This interpretation is supported by the similar prevalence of CKD stages 3–5 in the CARHES population aged 35–79 years and in our population aged 45–84: 2.89% vs 2.62%, respectively. The same study showed a slightly higher CKD stage 3–5 prevalence in female subjects (males 2.76%, females 3.03%). On the contrary, our results showed increased rates of CKD in males (males 3.28%, females 2.04%). According to a recent review that evaluates Italian experience in using administrative data to describe CKD epidemiology, there are very few studies on this topic [14]. In Canada, Tonelli et al. on behalf of the Alberta Kidney Disease Network used an algorithm based on hospitalization and ambulatory sensitive care condition system and found a prevalence of CKD in the population aged 18+ of 2.9%. When they added the estimated glomerular filtration rate and albuminuria, they found a prevalence of 20.4% [26, 27]. In the same ages we found a prevalence of 2.1% The heterogeneous methodology does not allow easy comparison of results [13, 14, 24]. In our study, we also tried to stratify CKD patients into groups characterized by higher and lower severity. Higher severity patients, defined by a combination of diagnostic codes of more advanced stages of CKD, need for renal replacement treatments and the prescription of drugs usually reserved for more advanced stages of disease, were found to represent slightly more than 20% of the whole population. Noteworthy, the fact that about 80% of the CKD population was defined as lower severity would suggest that our algorithm could effectively recognize less advanced stages of CKD where early interventions would be potentially more efficacious. Although we could not directly validate the accuracy of our stratification, it is interesting to note that the proportion of patients accessing all the main health systems explored increased from 5.1% (Fig. 2) in the overall CKD population to 24.0% (Fig. 3) in the higher severity group, an indirect proof of more severe disease demanding more extensive access to healthcare. In a previous study, the authors attempted to stratify patients into stages of CKD severity based on information obtained from informative systems such as ICD-10 codes, need for dialysis and prescription of drugs [28]. The authors found that such criteria were not able to discriminate between patients with eGFR below or above 90 mL/min/1.73 m^2^, however the specificity and negative predictive value at a threshold of 60 mL/min/1.73 m^2^ were very good. It is conceivable that the criteria used in our study to differentiate between patients with higher and lower severity might have similar properties.

The strengths of this study are the population-level approach and the use of standardized methodology for record-linkage procedures across multiple sources [20–22]. Since most regions in Italy use the same infrastructure for health data collection, our algorithm could be applied and validated in other regional contests. Our algorithm is strengthened by the poor selection bias (all the citizens in Italy have access to healthcare independent of census and the informative resources are generated automatically, thus ensuring complete coverage of the area), reduced misclassification error regarding the chronicity of kidney, high specificity as proven by the link with the dialysis registry and a large follow-up time. The major limitation is the lack of a gold standard for the determination of renal function. Previous studies have validated their algorithm with GFR estimating equations based on serum creatinine [28]. However, eGFR alone (e.g. without information on markers of kidney damage such as proteinuria or hematuria) is a potential source of overestimation of CKD, especially in the elder population [28]. Studies based on health information systems that use a real gold-standard for renal function such as iohexol clearance are not feasible.

## Conclusions

In conclusion, our new integrated algorithm based on data from different Health Information Systems in the Lazio region seems to be a useful tool for estimating the prevalence of subjects with CKD. The regional government can use it to guide the development and implementation of evidence-based pathways of care for CKD patients. Health care professionals should consider carefully the higher prevalence of lower severity CKD, since referral to specialists and optimal management of early stages may reduce the risk of worsening symptoms and complications.

## Data Availability

Data related to the findings reported in our manuscript are available to all interested researchers upon reasonable request and with the permission of the Regional Department, because of stringent legal restrictions regarding the privacy policy on personal information in Italy (national legislative decree on privacy policy n. 196/30 June 2003). For these reasons our dataset cannot be made available on public data deposition.

## Abbreviations

95%CI: 95% Confidence Intervals
ATC: Anatomical Therapeutic Chemical Classification System
CKD: Chronic kidney disease
ESRD: End-stage renal disease
HDR: The Hospital Discharge Registry
ICD-9-CM: International Classification of Disease-Ninth Revision-Clinical Modification
OSSIS: Outpatient Specialist Service Information System
PHARM: Drug Dispensing Registry
SD: Standard deviation
TER: Ticket Exemption Registry
